# The ELECLA trial: A multicentre randomised control trial on outcomes of neoadjuvant treatment on locally advanced colon cancer

**DOI:** 10.1111/codi.16908

**Published:** 2024-02-16

**Authors:** Jorge Arredondo, Ana Almeida, Carmen Castañón, Carlos Sánchez, Amaya Villafañe, Patricia Tejedor, Vicente Simó, Jorge Baixauli, Javier Rodríguez, Carlos Pastor, Enrique Pastor, Jesús Fernández, María Beltrán, Irene Oliva, Isabel Cifrián, Tomás Elosua, Amor Turienzo, María Victoria Diago, Cristina Rodríguez, Carmen Magdaleno, María José Suárez, Luis Martínez, Concepción Álvarez, Inmaculada Matanza, Patricia Tejedor, Ana López, Vincenzo Vigorita, Marta Tasende, Manuel Muinelo, Luis García Flórez, Isabel Gallarín, Felipe García, José Gil, Isabel Prieto, Lucía Ceniceros, Ignacio Matos, Alicia Alvarellos, Ana Chopitea, Paloma Rodríguez, Alicia Ruiz de la Hermosa, Antonio Climent, Diego Provedo, Francisco Blanco, Natalia Suárez

**Affiliations:** ^1^ Department of General Surgery Clínica Universidad de Navarra, University of Navarra Pamplona Spain; ^2^ Institute of Health Research of Navarra (IdisNA) Pamplona Spain; ^3^ Department of Oncology University Hospital of León Leon Spain; ^4^ Department of General Surgery University Hospital of León Leon Spain; ^5^ Department of General Surgery University Hospital Gregorio Marañón Madrid Spain; ^6^ Department of General Surgery University Hospital Río Hortega Valladolid Spain; ^7^ Department of Oncology, Clínica Universidad de Navarra University of Navarra Pamplona Spain; ^8^ Department of General Surgery, Clínica Universidad de Navarra University of Navarra Madrid Spain

**Keywords:** locally advanced colon cancer, neoadyuvant chemotherapy, Survival

## Abstract

**Background:**

Colon cancer (CC) is a public health concern with increasing incidence in younger populations. Treatment for locally advanced CC (LACC) involves oncological surgery and adjuvant chemotherapy (AC) to reduce recurrence and improve overall survival (OS). Neoadjuvant chemotherapy (NAC) is a novel approach for the treatment of LACC, and research is underway to explore its potential benefit in terms of survival. This trial will assess the efficacy of NAC in LACC.

**Methods:**

This is a multicentre randomised, parallel‐group, open label controlled clinical trial. Participants will be selected based on homogenous inclusion criteria and randomly assigned to two treatment groups: NAC, surgery, and AC or surgery followed by AC. The primary aim of this study is to evaluate the 2‐year progression‐free survival (PFS), with secondary outcomes including 5‐year PFS, 2‐ and 5‐year OS, toxicity, radiological and pathological response, morbidity, and mortality.

**Discussion:**

The results of this study will determine whether NAC induces a clinical and histological tumour response in patients with CCLA and if this treatment sequence improves survival without increasing morbidity and mortality.

**Registration number:**

NCT04188158.

**What does this paper add to the literature?**

Neoadjuvant chemotherapy is increasingly tested and employed for the treatment of locally advanced colon cancer. ELECLA trial will assess the impact of this novel approach in terms of survival, safety, feasibility and efficacy. This is a pragmatic trial, which employs an oxaliplatin‐ and fluoropyrimidine‐based scheme for pMMR colon adenocarcinoma.

## INTRODUCTION

Colon cancer (CC) is a public health problem due to its high prevalence and increasing incidence in younger patient populations [1]. It is the leading cause of cancer‐related death among males aged 20–49 years, with an ascending trend in young women in the USA [2].

Based on current clinical practice, the mainstay treatment for locally advanced CC (LACC) (stage III and high‐risk stage II) is oncological surgery followed by adjuvant chemotherapy (AC), which aims to decrease the risk of locoregional and distant relapse and increase overall survival (OS) [3]. Based on previous experiences with other locally advanced gastrointestinal tumours, neoadjuvant chemotherapy (NAC) is a relatively new concept in the treatment of LACC [4, 5, 6].

There are several potential advantages of NAC in LACC, such as reduced tumour cell‐shedding during surgery, increased likelihood of R0 resection, reduced number of positive lymph nodes, control of systemic metastatic spread, and the ability to test tumour chemosensitivity in vivo [7, 8, 9, 10]. Furthermore, the use of NAC is independent of eventual surgical complications, thus avoiding AC delays and improving chemotherapy completion rates.

Theoretically, however, NAC might increase perioperative complications and mortality rates. It has been a concern that tumour growth during NAC, in the case of poor tumour response, could lead to local complications, such as bowel obstruction or perforation requiring emergency surgery or even metastatic disease development. Furthermore, over staging by current imaging methods may lead to the overtreatment of low‐risk patients [8].

Several studies have demonstrated the safety and efficacy of NAC, but a survival benefit is still unclear [11, 12, 13, 14]. NAC has recently been added to the American guidelines from the National Comprehensive Cancer Network (NCCN) as an alternative for selected LACC cases (TNM‐T4b tumours) [15], based on studies that revealed a significantly improved survival rate [7]. Therefore, it is of great interest to explore the potential benefit of neoadjuvant therapy in LACC, analysing whether the tumour's response translates into a survival benefit.

## METHODS

This trial has been registered at ClinicalTrials.gov (https://clinicaltrials.gov/ct2/show/NCT04188158). The protocol has been structured following the Standard Protocol Items: Recommendations for Interventional Trials (SPIRIT) guidelines [16].

### Study objectives

The primary objective of this study is to determine if a therapeutic scheme based on NAC, surgery, and AC (Intervention group) increases the 2‐year progression‐free survival (PFS) compared to the standard treatment, based on surgery followed by AC (Control group) for LACC.

The secondary objectives are as follows:

1. To determine if the intervention group regimen increases 5‐year PFS compared to the control group.
2. To determine if the intervention group increases 2‐ and 5‐year overall survival (OS) compared to the control group.
3. To compare postoperative morbidity and mortality in both groups.
4. To assess toxicity in both groups.
5. To assess the therapeutic compliance rate in both groups.
6. To assess the accuracy of the CT scan for the diagnosis of LACC in the control group.
7. To assess the accuracy of the CT scan to evaluate tumour response after NAC in the intervention group.
8. To validate the use of tumour response scales in CC.
9. To determine if the addition of oxaliplatin impacts the pathological response in the group of patients over 70 years of age receiving NAC.

### Study design

This is a randomised, multicentre, parallel‐group, open label, controlled clinical trial. Both treatment groups can be framed within the usual clinical practice, and the procedures associated with the study do not deviate significantly from it.

### Eligibility criteria

#### Inclusion criteria

Clinical: (1) Histological confirmation of colon adenocarcinoma; (2) Age > 18 years for both males and females; (3) Good performance status (Karnofsky >60%, ECOG <2); (4) Blood analytics: haemoglobin >10 g/dL, leucocytes >3,0 × 10^9^/L, platelets >100.000 × 10^9^/L, glomerular filtration >50 mL/min, and total bilirubin <25 micromol/L; (5) Absence of chemotherapy contraindication and (6) Written and oral informed consent.

Radiological: (1) LACC: T4 or T3 with >5 mm extramural invasion on CT scan; (2) With or without nodal invasion on CT scan; (3) Absence of any distant metastases (M0) and (4) Radiologically resectable disease.

Therapeutical: All patients who undergo elective surgery with curative intention, regardless of the technique: open, laparoscopic, or robotic approach.

#### Exclusion criteria

(1) Significant comorbidity, uncontrolled angina, and myocardial infarction in the last 6 months; (2) Personal history of other neoplasia in the last 5 years; (3) Uncontrolled infective disease; (4) Pregnancy or breastfeeding; (5) Peripheral neuropathy grade > 1; (6) Rectal cancer (<15 cm from the anal verge or below peritoneal reflection); (7) Distant metastases or peritoneal carcinomatosis; (8) Bowel obstruction; (9) Microsatellite instability; (10) Refusal to participate or to give written informed consent and (11) If the patient cannot understand the purpose of the study or comply with its procedures.

### Study setting

Those responsible for recruiting and selecting patients for inclusion in the research project belong to each centre's Multidisciplinary Committee on Colorectal Cancer. A recruitment period of 2 years is estimated. Toxicity, compliance rate, tumour response, and perioperative morbimortality rate will be assessed. Two‐ and five‐year OS and PFS will be calculated.

### Patient identification and consent

All patients are evaluated at the time of diagnosis through medical history, physical examination, complete blood test with tumour markers, colonoscopy with biopsy and analysis of microsatellite instability. The patients are examined using a multidetector CT scan with oral and intravenous contrast. The standard CT acquisition protocol was performed in the venous phase and a section of 1.5 mm width was performed. This same protocol is employed for assessing the response in the interventional arm. In addition, a molecular determination is recommended but not mandatory: K‐N‐RAS and BRAF.

Potential candidates will be informed of the possibility of participating in the study verbally and in writing. Those who agree to participate (with written consent) and pass screening with the inclusion and exclusion criteria will be randomly assigned to one of the following treatment groups: (1) Intervention group: NAC + surgery + AC, or (2) Control group: Surgery + AC.

### Randomisation

Centralised randomisation will be performed with a 1:1 ratio using a computer‐generated sequence. Patients will be randomised either to the interventional or control group after first obtaining their informed consent. Stratified randomisation is performed based on the radiological T and N stages into four groups: T3N‐, T3N+, T4N‐, and T4N+. The attending surgeon will then be informed of the patient's treatment group. When obtaining consent, the attending surgeon will not be aware of the assigned group, as the randomisation will be performed afterwards.

### Interventions

#### Intervention group

NAC will follow a scheme based on three cycles of XELOX (capecitabine 1000 mg/m^2^ days) 1–14 + oxaliplatin 130 mg/m^2^ on day (1) every 21 days, over the course of 9 weeks. Oncological surgery will take place 4 weeks after completing NAC. The XELOX scheme could be replaced by five cycles of FOLFOX, depending on the practice of each centre.

Given the inconclusive data on the addition of oxaliplatin in patients over 70 years old in the MOSAIC [17] and NSABP C‐07 studies [18], in this subgroup of patients or in those with some comorbidities which limit the use of oxaliplatin, such as nephropathy, three cycles of capecitabine alone could be used instead exceptionally.

AC will start 4–6 weeks after surgery based on five cycles of XELOX, according to the initial clinical staging. If this interval is exceeded, the use of complementary therapy is left to the researcher's discretion.

#### Control group

The indication for AC will be based on the pathological findings; it is usually recommended in stage III or high‐risk stage II tumours. The standard scheme is based on eight cycles of XELOX.

According to the investigator's criteria and the usual guideline of each centre, the XELOX scheme could be replaced by 12 cycles of FOLFOX. In patients over 70 years old, eight cycles of capecitabine alone could be used exceptionally.

**FIGURE 1:**
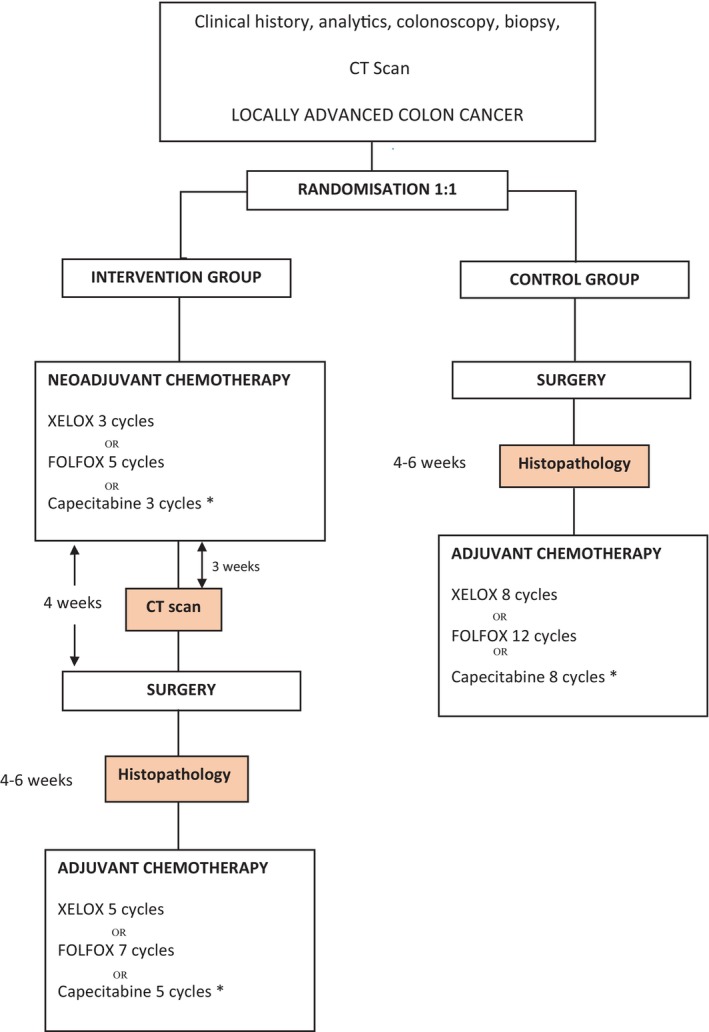
Flow diagram showing each stage of the trial. *Exceptionally in the group of patients with contraindication for oxaliplatin.

In this way, several therapeutic options are presented, collected in routine clinical practice, and in accordance with the most recent European, American, and Japanese reference guidelines [19] (Figure 1).

### Outcome parameters

The primary outcome of this trial will be 2‐year PFS. Follow‐up will be performed by Oncology and General Surgery Services, as per usual practice. During the first 2 years, a physical examination and assessment of tumour markers will be done every 3 months, whereas a thoracic‐abdominal‐pelvic CT scan will be performed every 6 months. One year after surgery, a colonoscopy will be performed. If no lesions are identified, repeat colonoscopy will be done after 5 years. If polyps are identified, then it will be requested after 2‐years. From the third to fifth postoperative year, physical examination, tumour markers, and CT scan will be performed every 6 months.

### Secondary outcome analysis

#### Tumour response grade

In the intervention group, the tumour response grade assessment will be radiologically assessed 3 weeks after NAC finishing by comparing pre‐ and post‐treatment TNM, tumour volume, and also according to the RECIST criteria [20]. Moreover, the pathological tumour response will be recorded, employing a modified Ryan scale, which is commonly used in rectal cancer [21].

#### Correlation between radiological and pathological findings

In the intervention group, the correlation between radiological and pathological response will be assessed to determine the predictive value of CT scan in evaluating tumour response grade. Information from the post‐treatment CT scan will be compared to the pathological findings. In the control group, the diagnostic accuracy of CT scan will be estimated by comparing the radiological basal stage to the pathological report. Sensitivity, specificity, and positive and negative predictive values will be calculated.

#### Postoperative morbidity and mortality

Postoperative morbidity and mortality will be graded according to the Clavien‐Dindo classification [22]. Transfusion requirements and long‐term outcomes will also be recorded, including incisional hernia and readmission. Chemotherapy toxicity will be recorded according to the CTCAE version 4.0.

### Statistical analysis and sample size calculation

Qualitative variables will be described using numbers and percentages. Quantitative variables will be described using the mean and standard deviation, or by median and interquartile range, for normally and distributed data, respectively. The Kolmogorov‐Smirnov test will be used to compare the normality of the variables. All randomised participants will be included in the population‐based intention‐to‐treat analysis. OS is defined as the time from surgery until death from any cause or follow‐up completion. PFS is defined as the time from the randomisation until local or distant recurrence or death.

PFS curves in the two treatment groups will be calculated using the Kaplan‐Meier method and compared using the Mantel Cox test. To determine the adjusted impact on OS and PFS, all clinically significant factors will be included in a Cox multiple regression model, and hazard ratio (HR) will be calculated with a 95% confidence interval. Statistical significance is set at *p* < 0.05. The SPSS version 15 software will be used for this purpose. Global statistical analysis will be performed by the main investigator.

This study is estimated to require 238 patients (119 per group, with a unilateral alpha type one error of 5%, power of 80%, and 5% of dropouts) to test the following hypothesis: Two‐year PFS of 75% and with an expected improvement of 15% in the neoadjuvant group, achieving a PFS of 90%.

### Participating centres and trial status

As of July 2023, 17 centres in Spain are currently included in the study, with more than 100 patients collected. The trial is expected to finish by June 2025.

This trial is supported by the European Society of Coloproctology (ESCP) and the Spanish Association of Coloproctology (AECP).

### Ethical approval

The study protocol has been approved by the local Research Ethics Committee and the Spanish Agency of Medicines and Medical Devices (AEMPS). Patients will be informed of the possibility of participating in the study and will need to provide written informed consent prior to enrolment. Patients will be able to withdraw their consent at any time without this affecting the medical care they will receive. The study will be performed in accordance with the Declaration of Helsinki. The main investigator at each participating centre will be responsible for patient recruitment and data recording; they will be audited by an independent data monitoring committee. Furthermore, periodic meetings or audits between the principal investigator at each centre and the study coordinator have been scheduled. Annual report of pharmacovigilance will be redacted, according to the Spanish Clinical Research Network.

The authors of this work declare that they have no conflict of interest in association with the present study, and they commit to publish the results even if they are not favourable to the hypothesis.

### Financial report

No financial compensation for participation in the study is foreseen for neither the patient nor the research team.

## DISCUSSION

For LACC, the standard treatment is based on early surgical resection, following oncological criteria, which is complemented with AC. The 5‐year survival rates for stage IIA and IIIC patients, respectively, are estimated to be 66.7% and 12.9%, according to data obtained from the Surveillance, Epidemiology, and End Result (SEER) [23]. Because of these results, the current treatment scheme cannot be considered as curative. The role of NAC in LACC is relatively new, but some recent studies have shown promising results [11, 14, 24, 25]. ELECLA is a phase II multicentre randomised trial which investigates the effectiveness of NAC in patients with LACC. This study evaluates PFS, because it is an accepted endpoint in studies evaluating adjuvant therapy which can correctly assess treatment efficacy and all‐cause mortality in each patient population. Moreover, it is also a suitable surrogate marker for 5‐year OS.

NAC is expected to achieve a tumour response, improve the R0 resection rate, and confirm its chemosensitivity [26, 27, 28]. For T4b tumours, oncological resection is the standard treatment, even considering complete en bloc multivisceral resection, which has been associated with higher postoperative morbidity and a lower percentage of R0 resection, ranging between 40% and 90% [29, 30]. In a recent meta‐analysis published in 2021, NAC was associated with significant margin‐negative resection rates and improved survival in T4 patients [7, 31]. As a result, NAC has been recommended for resectable T4 colonic tumours in the latest NICE (National Institute for Health and Care Excellence) and NCCN guidelines for colorectal cancer [15, 32]. Furthermore, NAC has been suggested to facilitate margin‐negative excision of LACC according to the new American Society of Colorectal Surgeons (ASCRS) guidelines for the management of colon cancer [33]. The recently published mature results from the multicentre FOxTROT phase III study are also in support of NAC; 6 weeks of preoperative chemotherapy in patients with LACC using a combination of cytotoxic drugs, including folinic acid, fluorouracil, and oxaliplatin, with or without panitumumab (a monoclonal antibody), was associated with a higher percentage of free resection margins (94% vs. 89% in the control group, *p* < 0.001) and a 28% lower recurrence rate compared to controls (rate ratio = 0.72; *p* = 0.037) [25].

In locally advanced rectal cancer, the impact of a pathological complete response (PCR) has been assumed to be a predictor of improved outcomes, but this assumption is still debatable for LACC [34]. To address this, Change et al. conducted a prospective observational study of 60 LACC patients who received neoadjuvant treatment and surgery; the PCR rate was higher in T3–T4a versus T4b patients, and PCR significantly improved patient OS [26]. The recent results of the OPTICAL trial, presented at the ASCO 2022 meeting, showed that NAC induced a 7% PCR rate and 20% tumour downstaging rate (pT0‐2N0M0) [35]. Significant tumour regression was also reported in the PRODIGE 22, a French phase II multicentre trial, which randomised 104 LACC patients to curative resection followed by AC or NAC followed by surgery and AC. Patients that underwent neoadjuvant treatment were more likely to achieve tumour remission grades 1–2 (44% vs. 8%, *p* < 0.001) [36]. Moreover, the PCR rate in the FOxTROT trial was 4%, and 62% of patients had some kind of regression. These findings are consistent with the previous published literature [14, 28], but there was no evidence that panitumumab improved the efficacy of NAC in patients with RAS wild‐type tumours [25]. This same trial demonstrated a 5‐year recurrence rate of 30% for those patients with no regression versus 0% in those with PCR, thus highlighting the importance of assessing the correlation between PCR and therapeutic outcomes [25]. These points should be considered when choosing and guiding adjuvant treatment, maybe in combination with the detection of minimal residual disease by circulating tumour DNA [37].

Patient selection and their corresponding therapeutic strategy are determined by CT scan. Several studies suggest that preoperative staging has limited accuracy [38, 39, 40], whereas other support its impact on survival [41, 42]. A significant concern raised from the data collected in these studies is whether preoperative CT staging is accurate enough to avoid overtreatment for low‐risk patients who otherwise would not require NAC or AC. In the preliminary results of the FOxTROT study, only 7% of T2N0 tumours were misclassified [8], although, in the results, 24% of control patients did not meet the criteria for AC [25]. Meanwhile, a Spanish study evaluated the accuracy of CT after NAC, and over staging was observed in 9.1% of cases [43]. In another study published by Huh et al. with more than 500 patients, CT had accuracies of 79.1% and 73.3%, respectively, for T and N staging. Radiological staging was also found to be an independent predictor of long‐term survival and was recommended for selecting patients with CC participating in clinical trials with NAC [42]. Magnetic resonance imaging allows a better soft tissue discrimination and diffusion‐weighted imaging and may become an interesting complementary tool for the selection of patients [44].

Another drawback is the possibility of continued tumour growth despite neoadjuvant treatment. In the FOxTROT trial, 4.3% of patients that received NAC required emergency surgery due to obstruction during preoperative treatment [34]. According to a single‐arm randomised phase II study with 47 patients, none had progression while receiving CapeOX (oxaliplatin plus capecitabine) [45]. Consistent results were observed in another study with 65 LACC patients, with a tumour volume reduction of 62.5%, without any case of progression [14]. In patients that undergo surgery upfront, some factors may encourage the development of micrometastasis and tumour progression, such as the stimulation of growth factors associated with the procedure and immunosuppression during the early postoperative period. In a recent propensity score matching analysis, none of the patients experienced progression, and the incidence of distant recurrence was significantly reduced [46]. NAC could control these potential factors, thereby improving the efficacy of cancer surgery through local downstaging and elimination of circulating tumour cells, potential micrometastases, and lymph node metastases [46, 47].

Although colonic obstruction is an exclusion criterion for NAC in our study, a diverting stoma or a self‐expanding metallic stent, as an alternative to emergency resection, enables improved local and distant tumour staging and the possibility of offering NAC, with its potential benefits [48, 49, 50].

Microsatellite instability is another exclusion criterion in our study. In the FOxTROT trial, the response to NAC was significantly lower in patients with deficient mismatch repair (dMMR) versus proficient mismatch repair (pMMR) tumours (7% vs. 23% moderate or greater histological tumour regression; *p* < 0.001). In addition, reductions in 2‐year recurrence were seen only in pMMR tumours, with no apparent benefit in dMMR tumours [25]. Furthermore, in a pilot study, neoadjuvant immunotherapy in LACC has shown promising results in dMMR tumours, with a 100% response rate [51]. This novel strategy has not yet been implemented in clinical practice or in our study. Larger studies and longer follow‐up are required. In our trial, we have chosen a neoadjuvant fluoropyrimidine‐oxaliplatin regimen without any monoclonal antibodies, as the latter was recently demonstrated to be ineffective in the preoperative setting [25]. Due to its increased toxicity, Irinotecan has not been considered for addition [52]. The rationale for offering three cycles of XELOX, rather than one or two, was to try to achieve maximum response; and rather than more cycles, to minimise the risk of tumour progression, in case of lack of response. The authors of the ELECLA trial have tried to offer a very pragmatic neoadjuvant scheme, looking for a high complementation rate in a real‐world scenario. Consequently, in the control arm, we have decided not to administrate adjuvant chemotherapy in the group of patients with pathological stage II without bad prognostic factors in order to avoid overtreatment, although it might be a bias.

The favourable tumour response to NAC warrants appropriate oncological control, but also results in a lower perioperative morbidity rate, contrary to previous assumptions that it renders patients less fit for surgery. In the FOxTROT trial, NAC did not increase surgical morbidity, but rather, less complications requiring emergency reoperation were observed [25]. A recent Dutch nationwide population‐based study found no significant difference in major complications and OS, which further supports the safety and feasibility of NAC [10]. These promising results, and those including neoadjuvant tolerance, are supported by other similar studies [14, 36, 45, 46, 53, 54]; even in adverse contexts, such as perforated CC [55].

All the initial experiences in the use of NAC for LACC support its feasibility, including a specifically directed study assessing the real‐life experience of implementing FOxTROT in other hospitals. This study also highlights the importance of a multidisciplinary committee [56]. Recent meta‐analyses have indicated that neoadjuvant treatment improves long‐term surgical and oncological outcomes [28, 57, 58]; but the final role of NAC in LACC in the therapeutic guidelines remains unresolved [24, 44, 59, 60, 61, 62]. This is the reason why the authors consider this topic worthy of continued research, as revealed by the ongoing FOxTROT2 and FOxTROT3, OPTICAL or NeoCol trials [63, 64, 65].

## AUTHOR CONTRIBUTIONS

**Jorge Arredondo:** Conceptualization; methodology; data curation; investigation; supervision; funding acquisition; visualization; project administration; resources; writing – original draft; writing – review and editing; software. **Ana Almeida:** Conceptualization; data curation; writing – original draft; writing – review and editing; validation. **Carmen Castañón:** Conceptualization; data curation; supervision; project administration; writing – original draft; writing – review and editing. **Carlos Sánchez:** Data curation; writing – review and editing. **Amaya Villafañe:** Data curation; writing – review and editing. **Patricia Tejedor:** Conceptualization; writing – original draft; writing – review and editing; formal analysis. **Vicente Simó:** Data curation; writing – original draft; writing – review and editing. **Jorge Baixauli:** Conceptualization; data curation; writing – review and editing. **Javier Rodríguez:** Writing – review and editing; data curation; conceptualization. **Carlos Pastor:** Writing – review and editing; writing – original draft; methodology; conceptualization.

## FUNDING INFORMATION

This trial is granted by the Spanish Society of Surgery (AEC), the Spanish Association of Coloproctology (AECP) and the Gerencia Regional de Salud de Castilla y León (SACYL).

## CONFLICT OF INTEREST STATEMENT

The authors of this work declare that they have no conflict of interest in association with the present study.

## ETHICS STATEMENT

This study was reviewed and approved by the Ethics Committee. Ethics Committee name: Comité Ético de Investigación Clínica de León.

## TRIAL REGISTRATION

EudraCT no.: 2016‐002970‐10. AEMPS no.: 16‐0553.

## ClinicalTrials.gov IDENTIFIER

NCT04188158.

## PROTOCOL VERSION

3, 1 July 2018.

## TRIAL SPONSOR

Jorge Arredondo Chaves. Clínica Universidad de Navarra, University of Navarra. Department of General Surgery, Av. Pío XII 36, 31008 Pamplona, Spain. jarredondo@outlook.es.

## Data Availability

The datasets generated and analysed during the present study are available from the corresponding author upon reasonable request.

## Appendix

Collaborators: Hospital Universitario de León: *Surgery*: Enrique Pastor, Jesús Fernández, María Beltrán, Irene Oliva, Isabel Cifrián, Tomás Elosua, Amor Turienzo, María Victoria Diago; *Radiology*: Cristina Rodríguez, Carmen Magdaleno, María José Suárez, Luis Martínez; *Pathology*: Concepción Álvarez, Inmaculada Matanza. Hospital Gómez Ulla, Madrid: *Surgery*: Patricia Tejedor. Hospital Universitario de Burgos: *Oncology*: Ana López. Hospital Universitario Álvaro Cunqueiro, Vigo: *Surgery*: Vincenzo Vigorita. Hospital Universitario Son Llàtzer, Palma de Mallorca: *Surgery*: Marta Tasende. Hospital Universitario Lucus Augusti, Lugo: *Surgery*: Manuel Muinelo. Hospital Universitario Central de Asturias, Oviedo: *Surgery*: Luis García Flórez. Hospital Universitario de Badajoz: *Surgery*: Isabel Gallarín. Hospital Universitario Nuestra Señora del Prado, Talavera de la Reina: *Surgery*: Felipe García. Hospital Universitario Virgen de la Arrixaca, Murcia: *Surgery*: José Gil. Hospital Universitario La Paz, Madrid: *Surgery*: Isabel Prieto. Clínica Universidad de Navarra, Madrid: *Oncology*: Lucía Ceniceros, Ignacio Matos; *Surgery*: Alicia Alvarellos. Clínica Universidad de Navarra, Pamplona: *Oncology*: Ana Chopitea. Hospital Universitario Río Hortega, Valladolid: *Surgery*: Paloma Rodríguez. Hospital Universitario Infanta Leonor, Madrid: *Surgery*: Alicia Ruiz de la Hermosa. Hospital Ribera Salud POVISA: Surgery: Antonio Climent, Diego Provedo. Hospital Universitario de Salamanca: *Surgery*: Francisco Blanco. Hospital Universitario de Ferrol: *Surgery*: Natalia Suárez.
